# Radiography may not be accurate in assessing acute ankle sprains in children

**DOI:** 10.1186/s13018-025-05480-1

**Published:** 2025-01-22

**Authors:** Xiongtao Li, Xiantao Shen, Si Wang, Jie Sun, Zhaoting Liu

**Affiliations:** 1 Department of Orthopedics Surgery, Tongji Medical College, Wuhan Children’s Hospital, Huazhong University of Science & Technology, No. 100 Hong-Kong Road, Jiang’an District, Wuhan City, 430014 PR China; 2 Department of Ultrasound Imaging, Tongji Medical College, Wuhan Children’s Hospital, Huazhong University of Science & Technology, No. 100 Hong-Kong Road, Jiang’an District, Wuhan City, 430014 PR China; 3 Department of Gastrointestinal Surgery, The First Hospital of Wuhan City, No. 215 Zhong-shan Road, Qiaokou District, Wuhan City, Wuhan 430022 PR China

**Keywords:** Radiography, Ultrasonography, Acute ankle sprains, Children, Diagnosis

## Abstract

**Background:**

Acute ankle sprains are among the most common injuries in children and are often associated with chondral avulsion fractures and ligament injuries. However, radiography may not be sufficiently accurate for assessing cartilage and ligament injuries in children. The primary purpose of this study was to evaluate the necessity of radiography in the diagnosis of acute ankle sprains in children. The secondary purpose was to assess whether ultrasonography can effectively improve the diagnostic accuracy of acute ankle sprains in children.

**Methods:**

We collected medical data from 78 children with acute ankle sprains who underwent both radiological and ultrasound examinations, 59 of whom also had ankle MRI results. The agreement between the radiographic and ultrasonographic findings in these 78 patients was assessed via Cohen’s kappa and McNemar tests. Using MRI results as the gold standard, the sensitivity, specificity, positive predictive value, and negative predictive value of radiography and ultrasonography were evaluated for the 59 patients who had MRI results. Cohen’s kappa and McNemar’s tests were also utilized to assess the reliability of radiography and ultrasonography in comparison to MRI.

**Results:**

Among the 78 children with acute ankle sprains, 29 did not show fractures on radiological examination, but fractures were detected via ultrasound examination. Among these 29 fractures, 20 were chondral avulsion fractures of the distal fibula, and 9 were avulsion fractures of the lateral talus process. The agreement between radiography and ultrasonography was fair (Kappa = 0.250), and the difference was statistically significant (*P* < 0.001). Using MRI as the gold standard, radiography resulted in 17 false-negative cases for lateral ankle fractures, with a sensitivity of only 47%. Ultrasonography produced only one false-negative case, achieving a sensitivity of 97%. Ultrasonography showed substantial agreement with MRI (Kappa = 0.797), whereas radiography showed fair agreement with MRI (Kappa = 0.384).

**Conclusions:**

Acute ankle sprains in children frequently result in a high incidence of chondral avulsion fractures. However, radiography may be inadequate for accurately diagnosing these fractures. Reliance on radiography alone may lead to a substantial number of false-negative cases. Compared with radiography, ultrasonography is highly accurate in the diagnosis of chondral avulsion fractures and ligament injuries in children. We believe that ultrasonography, as a noninvasive, radiation-free, and cost-effective dynamic diagnostic method, is particularly suitable for the early diagnosis of acute ankle sprains in children.

**Level of evidence:**

Level III; Diagnostic Study.

Acute ankle sprain is a common injury in children and may occur during daily life or sports activities [1, 2]. Paediatric acute ankle sprain may lead to lateral ankle ligament injury or avulsion fracture. Currently, radiology is the most commonly used diagnostic method. In the paediatric emergency department, up to 85–95% of children with ankle injuries require radiographic examination [3]. In many cases, paediatric ankle injuries are chondral avulsion fractures, however these cartilage lesions are not clearly revealed in radiographs [4, 5].

MRI scans can accurately detect ligament injuries and chondral avulsion fractures in the ankle [6]. However, children typically require sedation for MRI scans, which are time-consuming, expensive, and often difficult to perform quickly in many clinical settings [7]. Therefore, MRI may not be suitable as a routine diagnostic tool for acute ankle sprains in children. Additionally, Endele et al. reported that radiological examinations and ultrasound are sufficient for accurately diagnosing acute ankle sprains in children and that MRI does not provide additional value [8].

Ultrasound can clearly detect cartilage avulsion fractures and ligament injuries that might be overlooked [9]. Additionally, ultrasound for children does not require sedation, involves no radiation, is simple to perform, and can be completed quickly [9, 10]. Currently, ultrasound is widely used in the diagnosis of ankle injuries, but there is limited research investigating its diagnostic value in acute ankle sprains in children.

The primary purpose of our study was to assess the necessity of radiography in diagnosing acute ankle sprains in children. The secondary purpose was to evaluate whether ultrasonography can effectively improve the accuracy of diagnosing acute ankle sprains in children. We hypothesized that radiography is not necessary for diagnosing acute ankle sprains in children and that ultrasonography can effectively improve the accuracy of diagnosis.

## Methods

This study was approved by the Institutional Review Board of our institute. A spectrum of data from 78 children with acute ankle sprains who underwent both radiological and ultrasound examinations were collected between February 2023 and September 2024. The types of fractures, ligament injuries, and clinical presentations of the patients were recorded. Among these 78 patients, 59 had ankle MRI results. The inclusion criteria were as follows: aged 5 to 14 years; had a history of acute ankle sprain, with examinations completed within one week of injury; and had clear and standard radiographic and ultrasound examination results or MRI results. The exclusion criteria were as follows: the presence of fractures in other areas; Pain located at the level of the distal physis of the fibula; a Beighton ligament laxity score greater than 4; bilateral ankle sprains; and the presence of arthritis or other ankle joint diseases.

All patients completed radiological and ultrasound examinations. The ultrasound examinations were performed by senior physicians from the ultrasound imaging department. Both partial and complete avulsion fractures of the distal fibula and lateral tuberosity of the talus were considered positive, as were partial and complete tears of the anterior talofibular ligament and calcaneofibular ligament. The radiological and MRI examination results were jointly evaluated by a radiologist and a paediatric orthopaedic surgeon.

### Ultrasonography for the assessment of acute ankle sprain in children

Ultrasound examination of the lateral ankle was completed with the patient in the supine position. A high-frequency compact linear transducer of at least 10 MHz was used because the structures of the lateral ankle are superficial. To investigate lateral ankle fractures, the front end of the transducer was placed on the distal fibula and aligned with the longitudinal axis of the fibula. The front end was kept stationary and a pendulum motion was performed with the back end, covering the distal fibula and the lateral tuberosity of the talus to determine if there is an avulsion fracture of the lateral ankle.

To examine the anterior talofibular ligament in children, the transducer was placed on the distal fibula, with the front end positioned at the anterior medial edge of the distal fibula and the back end pointing between the second and third toes. Then, the transducer was moved downwards. Once the transducer reached the distal tip of the fibula, slight adjustments were made to visualize the talus. At this position, the anterior talofibular ligament appears as a homogeneously hypoechoic structure. A normal anterior talofibular ligament is seen as a continuous, compact fibrillar structure extending from the fibula to the talus along the long axis.

### Statistical analyses

The descriptive variables are reported as frequencies (percentages). The consistency between the radiological and ultrasound examination results for these 78 patients was assessed via Cohen’s kappa and McNemar tests. For the 59 patients with radiological, ultrasound, and MRI findings, the MRI results were used as the “gold standard” to evaluate the sensitivity, specificity, positive predictive value, and negative predictive value of radiography and ultrasonography. Cohen’s kappa and McNemar tests were also used to assess the reliability of radiological and ultrasound examinations compared with MRI. Kappa values were interpreted via the criteria of Landis and Koch [11]: 0.01 to 0.20, slight agreement; 0.21 to 0.40, fair agreement; 0.41 to 0.60, moderate agreement; 0.61 to 0.80, substantial agreement; and 0.81-1.00, excellent agreement. Significance was set at *P* < 0.05. All analyses were performed using SPSS software (version 27.0; IBM).

## Results

**Fig. 1:**
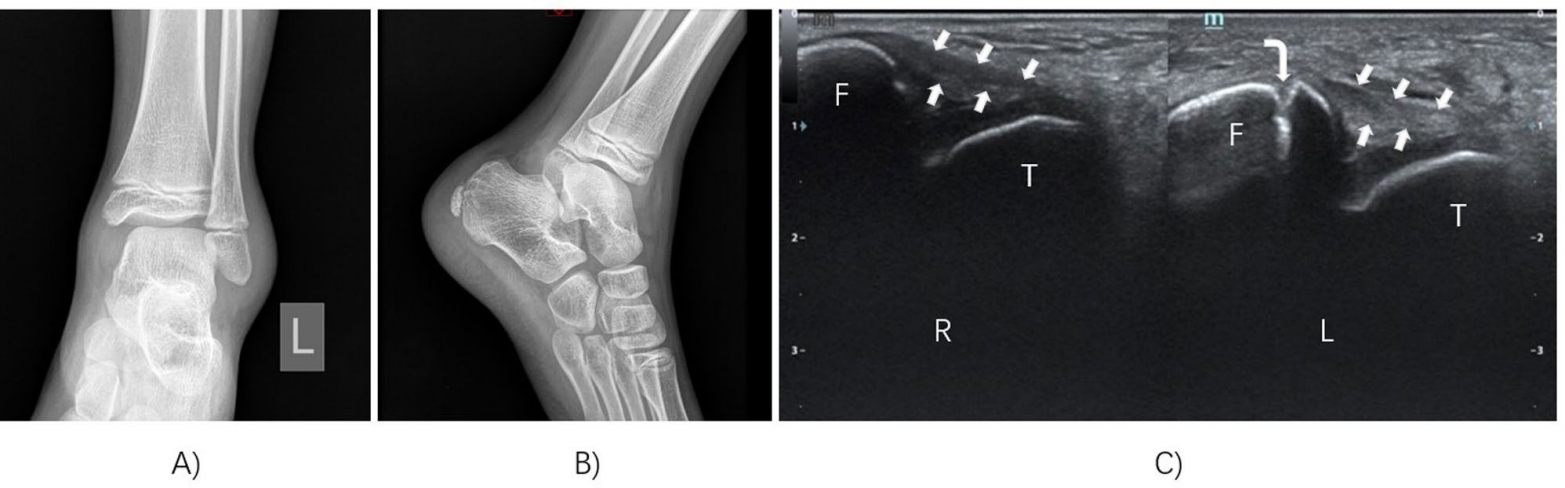
Chondral avulsion fractures of the distal fibula were negative on radiography but positive on ultrasonography. (**A**) Anteroposterior X-ray radiographs of the ankle. (**B**) Lateral X-ray radiographs of the ankle. (**C**) Ultrasound examination of the ankle was performed and compared with the contralateral side. *R*, right; *L*, left; *F*, fibula; *T*, talus; arrowhead, anterior talofibular ligament; right angle arrow, chondral avulsion fracture of the distal fibula

We found that among the 78 children with acute ankle sprains, radiological examination revealed 16 cases (21%) of lateral ankle fractures, whereas ultrasound examination revealed 43 cases (55%) of lateral ankle fractures. There were 29 patients who did not have fractures on radiological examination, but these fractures were identified via ultrasound examination (Table 1). Among these 29 fractures detected by ultrasonography, 20 were chondral avulsion fractures of the distal fibula, and 9 were avulsion fractures of the lateral talus process (Fig. 1). The kappa value was 0.250, indicating fair agreement between radiography and ultrasonography. The difference was statistically significant (*P* < 0.001).

**Table 1:** Radiography and ultrasonography for diagnosing lateral ankle fractures in children

		Radiography	
		+	-
US	**+**	14	29
	**-**	2	33

US: Ultrasonography

Among the 59 patients with MRI, radiological, and ultrasound results. We found that radiography yielded 17 false-negative results for lateral ankle fractures in children with acute ankle sprain, resulting in a sensitivity of only 47%. In contrast, ultrasound examination revealed only one false-negative case, with a sensitivity of 97% (Table 2). Ultrasonography showed substantial agreement with MRI (Kappa = 0.797), whereas radiography showed fair agreement with MRI (Kappa = 0.384). The difference in results between radiography and MRI was statistically significant (*P* < 0.001).

**Table 2:** The reliability of radiography and ultrasonography was evaluated based on MRI as the “gold standard”

	TP/TN(*n*)	FP/FN(*n*)	Sensitivity	Specificity	PPV	NPV	Youden’s index	Kappa value	*P* value*
Fractures of lateral ankle									
Radiography	12/29	1/17	41%	97%	92%	63%	0.38	0.384	< 0.001
US	28/25	5/1	97%	83%	85%	96%	0.80	0.797	0.219
Ligament injuries assessed by US									
ATFL	22/34	2/1	96%	94%	92%	97%	0.90	0.894	1.000
CFL	5/52	1/1	83%	98%	83%	98%	0.81	0.814	1.000

^*^ The *P* value is the result of the McNemar test. TP: True Positive; TN: True Negative; FP: False Positive; FN: False Negative; PPV: Positive Predictive Value; NPV: Negative Predictive Value; US: Ultrasonography; ATFL: Anterior Talofibular Ligament; CFL: Calcaneofibular Ligament

**Fig. 2:**
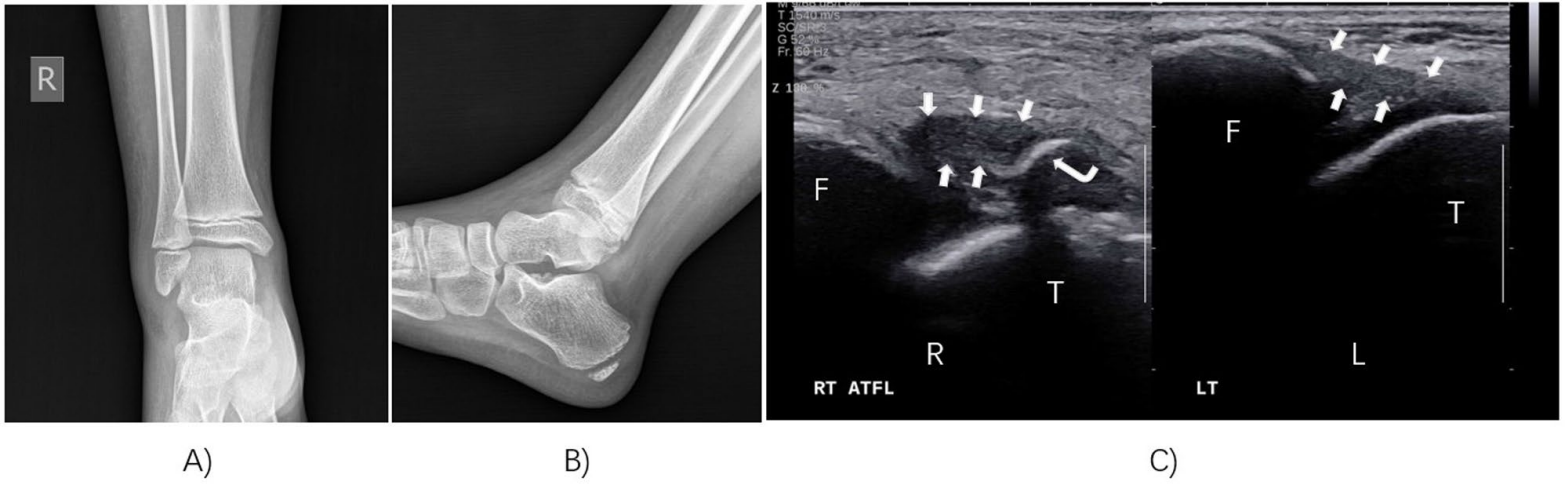
Chondral avulsion fracture of the lateral talus process was negative on radiography but positive on ultrasonography. (**A**) Anteroposterior X-ray radiographs of the ankle. (**B**) Lateral X-ray radiographs of the ankle. (**C**) Ultrasound examination of the ankle was performed and compared with the contralateral side. *R*, right; *L*, left; *F*, fibula; *T*, talus; arrowhead, anterior talofibular ligament; right angle arrow, chondral avulsion fracture of the lateral talus process

**Fig. 3:**
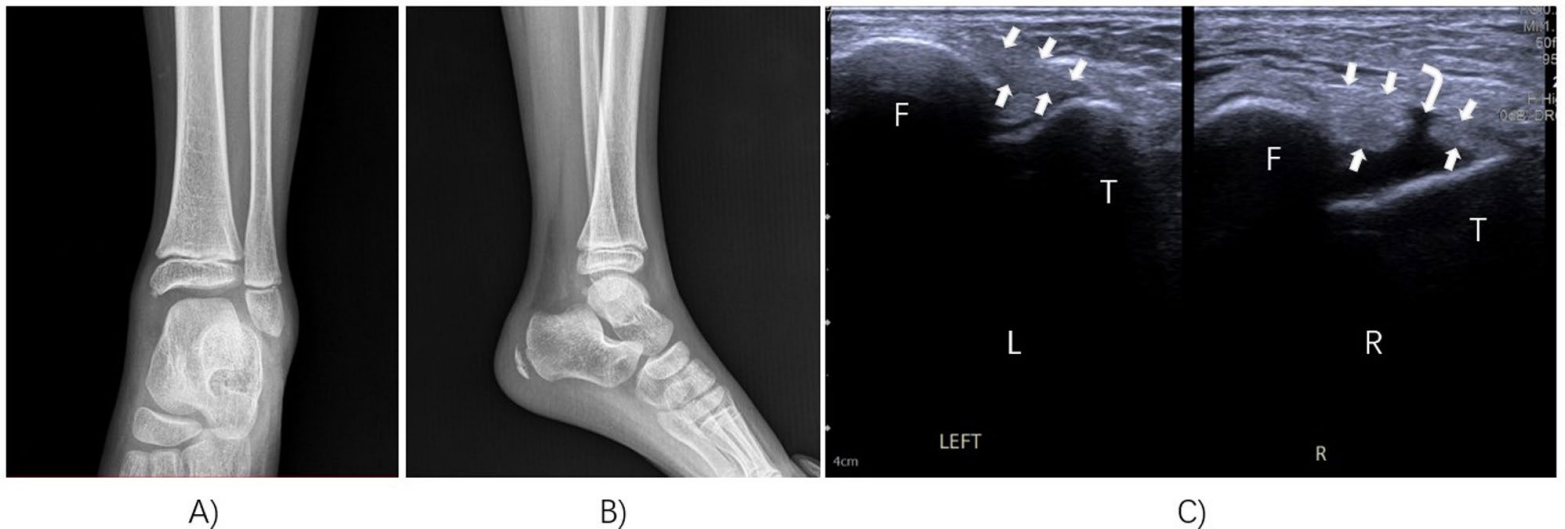
Anterior talofibular ligament injury was positive on ultrasonography. (**A**) Anteroposterior X-ray radiographs of the ankle. (**B**) Lateral X-ray radiographs of the ankle. (**C**) Ultrasound examination of the ankle was performed and compared with the contralateral side. *R*, right; *L*, left; *F*, fibula; *T*, talus; arrowhead, anterior talofibular ligament; right angle arrow, the site of injury to the anterior talofibular ligament

Ultrasound and MRI can both detect ligament injuries in the lateral ankle following acute ankle sprain in children. In our study, we found that the sensitivity of ultrasonography for detecting anterior talofibular ligament (ATFL) injuries was 96%, and the specificity was 94% (Fig. 2). There was excellent agreement with the MRI results (Kappa = 0.894), and the difference was not statistically significant. Similarly, the sensitivity of ultrasound examination for detecting calcaneofibular ligament (CFL) injuries was 83%, and the specificity was 98%. There was also excellent agreement with the MRI results (Kappa = 0.814) (Table 2) (Fig. 3).

## Discussion

Acute ankle sprain is one of the most common injuries to the lower extremities, with an incidence rate of 11.88%, and it occurs more frequently during sports activities [5, 12, 13]. Acute ankle sprain in children is often accompanied by lateral ankle ligament injuries and avulsion fractures of the lateral ankle [14, 15]. The anterior drawer test and inversion stress test are classic methods for examining whether the anterior talofibular ligament (ATFL) is injured, but it is usually difficult for children with acute sprains to complete these tests [16, 17]. Surgeons typically use the “Ottawa Ankle Rules” to determine whether radiological examination is necessary [18]. However, in cases of acute ankle sprain in children, lateral ankle fractures are often avulsion fractures involving cartilage, and radiography of the ankle usually cannot adequately demonstrate cartilage damage.

Schutze and Maas reported that in children with ankle sprains, one-third of avulsion fractures cannot be identified through radiography [19]. However, Haraguchi et al. reported that 76% of avulsion fractures in children can be detected via radiography, as the authors reported that most chondral avulsion fractures are associated with bony tissue avulsion, which can be detected via radiological imaging [20]. In our study, we found that while some children with ankle sprains do have bony avulsions (41%) based on X-ray results, simple chondral avulsion fractures (59%) are more common. For this reason, a total of 17 false-negative results were produced by ankle radiography in our study, with a sensitivity of only 41% and a negative predictive value of 63%.

Ultrasound examination has unique advantages in detecting cartilage injuries. Simanovsky et al. [21] found that ultrasonography has high sensitivity and specificity in the diagnosis of chondral avulsion fractures of the ankle. In our study, we compared 79 cases of lateral ankle fractures via radiography and ultrasonography. We found that 29 cases of avulsion fractures of the lateral ankle were detected via ultrasonography but were missed via radiography. Interestingly, while it is commonly believed that avulsion fractures of the lateral ankle are typically distal fibular chondral avulsion fractures, our study revealed that 31% (9 out of 29) of the cases were actually avulsion fractures of the lateral talus process. However, it is important to note that pain at the level of the distal fibular physis should not be overlooked, as a physeal injury can lead to epiphysiolysis, which requires a different diagnostic and therapeutic approach.

In this study, 59 cases were evaluated with MRI as the gold standard, and the sensitivity of ankle ultrasound in diagnosing lateral ankle fractures reached 97%, whereas the sensitivity of radiological diagnosis for lateral ankle fractures was only 41%. This indicates that if the presence of lateral ankle fractures in children with acute ankle sprain is determined only based on radiography results, a significant number of false-negative cases may occur. Additionally, five false-positive cases were identified in the ultrasonography diagnosis in this study, which may be due to the avulsion fractures being too small to be detected by MRI or possibly due to oedema at the fracture site, leading to missed diagnoses in the MRI results. If avulsion fractures are difficult to definitively diagnose during ultrasound examination, dynamic stress ultrasonography can be performed, and partial stress can be applied to the fracture site to assess the stability of the fracture, which is a unique advantage of ultrasonography [22]. Ultrasound examination can assist orthopaedic surgeons in making more accurate diagnoses, but this does not imply that the treatment plan will be altered. In fact, most acute ankle sprains in children achieve good treatment outcomes without requiring surgical intervention.

The lateral ligaments of the ankle in children are often injured following acute sprains, with over 80% of ankle sprain patients experiencing anterior talofibular ligament injury [16, 23]. However, ankle radiography cannot accurately determine whether the anterior talofibular ligament is damaged. Since both the anterior talofibular ligament and the calcaneofibular ligament are located relatively superficially, ultrasonography of the ankle can clearly detect injuries to these ligaments [24–26]. Hosseinian et al. reported in their study of adult ankle sprains that ultrasonography for diagnosing anterior talofibular ligament injuries achieved 100% sensitivity, specificity, positive predictive value, and negative predictive value, with a kappa value of 1 [24]. Our results are similar: ultrasonography yielded two false-positive cases and one false-negative case, with a sensitivity of 96%, specificity of 94%, positive predictive value of 92%, negative predictive value of 97%, and a kappa value of 0.894.

Unlike previous studies on adult ankle ligament injuries, we found that only 5 cases involved calcaneofibular ligament injuries in children, which is significantly fewer than the number of anterior talofibular ligament injuries [27]. However, the accuracy of musculoskeletal ultrasound in diagnosing calcaneofibular ligament injuries is still high, with a sensitivity of 83%, a specificity of 98%, and a kappa value of 0.814. Our study results show that the agreement between ultrasound and MRI in diagnosing anterior talofibular ligament and calcaneofibular ligament injuries is very high, indicating that ultrasonography can be a reliable alternative to MRI.

The main limitation of this study was the lack of interobserver and intraobserver reliability of the ultrasound examination results, as these results may be related to the experience of the sonographer [4]. However, in our study, the interpretation of the ultrasound results was performed by a senior sonographer with extensive experience in musculoskeletal ultrasound, which should largely mitigate the bias that might arise from an inexperienced sonographer.

## Conclusions

Acute ankle sprains in children often result in avulsion fractures of the lateral ankle cartilage and injuries to the lateral ankle ligaments. However, radiography has difficulty accurately determining the presence of chondral avulsion fractures and ligament injuries. When compared with ultrasound and MRI examination results, radiography may lead to a significantly higher number of false-negative cases.

Ultrasonography is a noninvasive, radiation-free, and cost-effective dynamic diagnostic method. Compared with radiography, ultrasonography is particularly suitable for the diagnosis of chondral avulsion fractures of the lateral malleolus in children. Compared with ankle MRI, ultrasonography also shows very high accuracy in diagnosing acute injuries to the anterior talofibular ligament and calcaneofibular ligament in children. Therefore, we believe that ultrasonography is highly suitable for the early diagnosis of acute ankle sprains in children.

## Data Availability

No datasets were generated or analysed during the current study.
